# Preoperative Serum Total IgE Category and 6-Month Tympanometric Status After Type I Tympanoplasty for Chronic Otitis Media

**DOI:** 10.3390/audiolres16050146

**Published:** 2026-09-28

**Authors:** Dan Chen, Yingying You, Luoying Yang, Jianyan Wang, Chengyu Ge, Peng Li, Yubin Chen

**Affiliations:** 1 Department of Otolaryngology Head and Neck Surgery, The Third Affiliated Hospital of Sun Yat-sen University, Guangzhou 510630, China; chend67@mail.sysu.edu.cn (D.C.); yangly9@mail.sysu.edu.cn (L.Y.);; 2 Department of Otolaryngology Head and Neck Surgery, The Eighth Affiliated Hospital of Sun Yat-sen University, Shenzhen 518000, China; youyy9@mail.sysu.edu.cn

**Keywords:** serum total IgE, chronic otitis media, type I tympanoplasty, tympanometry, Eustachian tube, mastoid pneumatization

## Abstract

**Objective:** To evaluate whether preoperative serum total IgE, analyzed categorically and continuously, was associated with 6-month postoperative tympanometric status after type I tympanoplasty for chronic otitis media. Tympanic membrane closure and audiometric outcomes were also examined. **Methods:** This retrospective study included 171 operated ears from 171 unique patients with chronic otitis media who underwent type I tympanoplasty. The categorical analysis used the institutional assay upper reference limit of 60 IU/mL; a separate analysis treated IgE as a log2-transformed continuous variable. Tympanic membrane closure, tympanometry, and audiometry were assessed at 6 months. Abnormal tympanometric status was defined as a measurable peak pressure < −50 daPa or a type B tympanogram. Multivariable logistic regression evaluated factors associated with abnormal tympanometric status. **Results:** Of 171 patients, 101 had serum total IgE ≤ 60 IU/mL and 70 had IgE > 60 IU/mL. Abnormal 6-month tympanometric status occurred in 22/89 (24.7%) and 29/61 (47.5%) ears, respectively (*p* = 0.005). Tympanic membrane closure was 96/98 (98.0%) versus 61/68 (89.7%) (*p* = 0.033). A 6-month ABG ≤ 20 dB occurred in 87/94 (92.6%) versus 54/64 (84.4%) (*p* = 0.121). In complete-case multivariable analysis, IgE > 60 IU/mL (OR, 3.46; 95% CI, 1.56–7.70; *p* = 0.002) and poor mastoid pneumatization (OR, 4.68; 95% CI, 2.15–10.21; *p* < 0.001) were associated with abnormal tympanometric status. In the separate continuous analysis, each doubling of IgE was associated with higher adjusted odds of abnormal tympanometric status (OR, 1.25; 95% CI, 1.03–1.52; *p* = 0.023). **Conclusions:** Preoperative serum total IgE was associated with abnormal 6-month tympanometric status in both categorical and continuous analyses. The 60 IU/mL cutoff was an assay-specific reference limit rather than a validated postoperative risk threshold. The findings do not establish predictive or prognostic utility, clinically defined allergy, or an allergy-mediated causal mechanism.

## 1. Introduction

Chronic otitis media (COM) with tympanic membrane perforation remains a common otologic disorder and a major cause of recurrent otorrhea and conductive hearing loss [1]. Recent epidemiologic work continues to show that chronic suppurative otitis media contributes substantially to the global burden of ear disease and hearing impairment [2]. Type I tympanoplasty is a standard reconstructive procedure for restoring tympanic membrane integrity, protecting the middle ear, and improving hearing. Modern microscopic and endoscopic techniques achieve favorable anatomical and audiometric results in many patients, yet postoperative outcomes remain variable [3,4].

Many studies have attempted to identify predictors of tympanoplasty success. Traditional factors include perforation size, wet versus dry ear status, eustachian tube dysfunction (ETD), middle-ear mucosal status, mastoid pneumatization, graft material, surgical approach, and surgeon experience [5,6]. However, the previous literature remains heterogeneous. Some studies support a role for eustachian tube function or mastoid aeration, whereas others show acceptable results despite abnormal preoperative findings [7,8,9]. Similarly, wet ear status has historically been considered a risk factor, but recent studies have reported comparable closure and hearing outcomes between dry and wet ears [10,11,12,13]. These observations suggest that some traditional variables may describe preoperative disease phenotype rather than independently determine postoperative recovery.

Immunoglobulin E (IgE) has long been investigated in middle-ear disease, particularly in otitis media with effusion (OME) and eosinophilic otitis media. Previous studies have evaluated both serum total IgE and IgE within middle-ear effusion, but the findings have been heterogeneous. Some studies found no significant difference in serum total IgE between patients with OME and controls, whereas others reported associations between serum IgE and IgE detected in middle-ear effusion [14,15,16]. Although serum total IgE does not necessarily reflect local middle-ear IgE activity or establish clinically relevant allergic disease, studies of eosinophilic otitis media have demonstrated high concentrations of total and antigen-specific IgE within middle-ear effusion and suggested that IgE may be produced locally in the middle-ear mucosa [17]. Eosinophilic otitis media is, however, a distinct clinical entity; therefore, these findings support biological plausibility but do not establish that the same mechanism operates in patients undergoing tympanoplasty for chronic otitis media.

An IgE-related pathway is nevertheless biologically plausible because the nasal mucosa, pharyngeal eustachian tube, and middle ear are anatomically and functionally connected. IgE-mediated inflammation of the nasal mucosa may cause edema and increased secretion around the pharyngeal Eustachian tube orifice, potentially impairing tubal opening, mucociliary clearance, and middle-ear pressure regulation. The recent EAACI position paper identifies allergy and IgE-mediated immunity as potential contributors to OME and Eustachian tube dysfunction, while emphasizing that the available evidence remains heterogeneous and does not support a uniform causal relationship [18]. In a prospective controlled study of patients with house dust mite-allergic rhinitis, objective tubomanometric abnormalities were more frequent than in controls, correlated with nasal obstruction and endoscopic inflammation, and improved after treatment with an intranasal glucocorticoid and an antihistamine [19]. Although these observations were not obtained in tympanoplasty patients, they provide clinical support for a potential link between an IgE-associated upper-airway inflammatory phenotype and impaired middle-ear pressure regulation, without implying that allergy is the sole or dominant cause.

This potential pathway is clinically relevant after tympanoplasty because successful tympanic membrane closure does not necessarily ensure restoration of middle-ear aeration and pressure regulation. Postoperative tympanometry provides an objective assessment of middle-ear status after reconstruction. Within the composite endpoint used in the present study, a measurable negative-pressure peak primarily reflects pressure dysregulation, whereas a type B tympanogram may represent a different or more complex middle-ear condition; these two components should therefore not be assumed to represent an identical postoperative physiological state. Because postoperative tympanometric outcomes may remain variable even after conventional anatomical and functional factors are considered, serum total IgE was examined as a routinely available but nonspecific laboratory measure that might reflect an IgE-associated host characteristic not captured by standard otologic variables. Importantly, serum total IgE cannot diagnose allergic rhinitis, directly measure local middle-ear inflammation, or establish an allergy-mediated causal mechanism. The present analysis was therefore intended to test a biologically informed and clinically relevant association, rather than to validate serum total IgE as a prognostic biomarker or to establish a clinical cutoff for postoperative risk stratification.

Accordingly, the present study evaluated whether preoperative serum total IgE, analyzed using the institutional assay reference category and separately as a continuous measure, was associated with 6-month postoperative tympanometric status after type I tympanoplasty. Tympanic membrane closure and audiometric outcomes were evaluated as additional clinical endpoints.

## 2. Materials and Methods

### 2.1. Study Design and Patients

This retrospective study included 171 operated ears from 171 unique patients with COM who underwent type I tympanoplasty and had available serum total IgE data at the Department of Otolaryngology Head and Neck Surgery, the Third Affiliated Hospital of Sun Yat-sen University (January 2021 to December 2025). The study was approved by the Ethics Committee of the Third Affiliated Hospital of Sun Yat-sen University (approval Code: II2025-513-01, approval Date: 29 December 2025) and was conducted in accordance with the Declaration of Helsinki. Written informed consent was obtained from all participants or their legal guardians, as applicable.

### 2.2. Clinical Variables and Definitions

Continuous serum total IgE measurements were abstracted from the original laboratory records for all 171 patients. The 60 IU/mL threshold corresponded to the upper reference limit used by our hospital laboratory for the fluorescent enzyme-linked immunoassay, and patients were categorized as IgE ≤ 60 IU/mL or IgE > 60 IU/mL. Allergen-specific IgE, skin-prick testing, detailed nasal symptom scores, nasal examination findings, and antiallergic treatment data were not retained; therefore, the IgE category was not considered a diagnosis of allergic disease. Preoperative Eustachian tube function was assessed using the 10-point Eustachian Tube Score (ETS), which incorporates tubomanometry at 30, 40, and 50 mbar, the ability to perform the Valsalva maneuver, and the subjective perception of a clicking sound during swallowing [20]. In the protocol used for this cohort, an ETS ≤ 5 was classified as abnormal and an ETS > 5 as normal. Tympanic membrane perforations involving <50% of the membrane area were classified as small, whereas those involving ≥50% were classified as large. Ear status was classified as dry or wet according to the presence of preoperative otorrhea or inflammatory middle-ear secretion. Purulent and nonpurulent wet-ear phenotypes were not consistently separated in the retrospective records, and routine microbiological cultures were not obtained. Mastoid pneumatization was classified as well pneumatized or poorly pneumatized according to the preoperative clinical record and temporal-bone imaging assessment.

Pure-tone air-conduction and bone-conduction thresholds were summarized at 0.5, 1, 2, and 4 kHz. The air-bone gap (ABG) was calculated from the corresponding air- and bone-conduction averages at these frequencies. Postoperative audiometry was performed at 6 months. A postoperative ABG ≤ 20 dB was used as a conventional functional hearing endpoint, consistent with recent tympanoplasty studies [21].

Postoperative tympanometry was performed at 6 months. Type A tympanograms with a measurable peak pressure ≥ −50 daPa were considered normal [22]. Abnormal postoperative tympanometric status was defined as either a type C tympanogram with a measurable peak pressure < −50 daPa or a type B tympanogram. Because these components may represent different postoperative physiological states, type A, type B, and type C tympanograms were also analyzed separately.

Tympanic membrane closure was assessed by endoscopic examination at 6 months after surgery.

### 2.3. Surgical Procedure and Follow-Up

All procedures were performed endoscopically by the same senior otologic surgeon using an underlay type I tympanoplasty technique. A tragal cartilage–perichondrium composite graft was harvested from the operated ear, with perichondrium preserved on one surface of the cartilage. The tympanic membrane remnant was routinely thinned before graft placement. Laser was not used during tympanic membrane reconstruction. Concomitant Eustachian tube balloon dilation was performed in selected patients according to the preoperative clinical assessment and was recorded as a covariate.

### 2.4. Statistical Analysis

All data were statistically analyzed using SPSS version 27.0 (IBM Corp., Armonk, NY, USA). Continuous variables were expressed as mean ± standard deviation (SD), except serum total IgE, which was summarized as median and interquartile range because of its right-skewed distribution. Categorical variables were compared using the chi-square test or Fisher’s exact test, as appropriate. Quantitative variables were compared using Student’s *t*-test, and paired data were analyzed using the paired *t*-test. Multivariable logistic regression was used to identify factors associated with abnormal postoperative tympanometric status at 6 months. The primary model included age, sex, wet-ear status, perforation size, preoperative ETD, mastoid pneumatization, serum total IgE category, and concomitant balloon dilation. In sensitivity analyses, the binary IgE category was replaced by log2-transformed total IgE so that the odds ratio represented each doubling in IgE concentration. No missing-data imputation was performed; analyses of individual outcomes were based on available cases, and the multivariable models used complete cases. Because only two patients were younger than 18 years, an adult-only sensitivity analysis excluding these patients was performed. A two-sided *p*-value < 0.05 was considered statistically significant. To examine the composition of the composite tympanometric endpoint, exploratory component-specific logistic regression analyses compared type B with type A and type C with type A. Serum total IgE category and log2-transformed continuous IgE were evaluated in separate models using the same covariates as the primary analysis. Because of the limited number of component-specific outcomes, these analyses were considered exploratory.

## 3. Results

### 3.1. Clinical Characteristics

The cohort comprised 171 operated ears from 171 unique patients: 101 in the serum total IgE ≤ 60 IU/mL group and 70 in the >60 IU/mL group. The overall median serum total IgE was 51.0 IU/mL (interquartile range, 23.15–196.0 IU/mL). Mean age was approximately 43 years (range, 8–74 years); two patients were younger than 18 years (ages 8 and 15). Baseline age, sex, operated side, dry/wet ear status, perforation size, preoperative ETD, mastoid pneumatization, and preoperative hearing measures did not differ significantly between IgE categories (Table 1). Concomitant balloon dilation was more frequent in the IgE > 60 IU/mL group (40.0% vs. 24.8%, *p* = 0.044). Preoperative ABG was available in 169 ears, of which 67 (39.6%) had an ABG > 20 dB.

### 3.2. Overall Surgical Outcomes

Overall surgical outcomes are summarized in Table 2A,B. At 6 months, tympanic membrane closure was achieved in 157/166 (94.6%) ears, whereas 51/150 (34.0%) had abnormal tympanometric status. A 6-month ABG ≤ 20 dB was present in 141/158 (89.2%) ears. Air-conduction PTA, bone-conduction average, and ABG improved significantly from preoperative values (all *p* < 0.001; Table 2A,B).

Among the 150 ears with available 6-month tympanometry, 99 had a type A tympanogram, 19 had a type B tympanogram, and 32 had a type C tympanogram with a measurable peak pressure below −50 daPa. In the IgE ≤ 60 IU/mL group, the corresponding distribution was 67/89 (75.3%), 10/89 (11.2%), and 12/89 (13.5%), compared with 32/61 (52.5%), 9/61 (14.8%), and 20/61 (32.8%) in the IgE > 60 IU/mL group, respectively (overall *p* = 0.009; Table 3). The IgE > 60 IU/mL group also had a higher rate of the prespecified composite abnormal tympanometric endpoint (29/61 [47.5%] vs. 22/89 [24.7%], *p* = 0.005) and a lower tympanic membrane closure rate (61/68 [89.7%] vs. 96/98 [98.0%], *p* = 0.033). The proportion with a 6-month ABG ≤ 20 dB did not differ significantly (54/64 [84.4%] vs. 87/94 [92.6%], *p* = 0.121; Table 3).

Among the 63 ears with a preoperative ABG > 20 dB and available 6-month audiometry, 51 (81.0%) achieved a 6-month ABG ≤ 20 dB. This proportion did not differ significantly between the IgE > 60 IU/mL and ≤60 IU/mL groups (19/25 [76.0%] vs. 32/38 [84.2%], *p* = 0.517; Table 3).

### 3.3. Multivariable Analysis

Multivariable logistic regression for abnormal postoperative tympanometric status is shown in Table 4. Complete-case multivariable analysis included 147 ears. Serum total IgE > 60 IU/mL was independently associated with abnormal 6-month tympanometric status (OR, 3.46; 95% CI, 1.56–7.70; *p* = 0.002), as was poor mastoid pneumatization (OR, 4.68; 95% CI, 2.15–10.21; *p* < 0.001). Age, sex, wet-ear status, perforation size, preoperative ETD, and concomitant balloon dilation were not independently significant (Table 4). In the separate continuous-IgE analysis, the unadjusted OR for each doubling of IgE was 1.18 (95% CI, 1.00–1.40; *p* = 0.053). After adjustment for the same covariates used in the primary model, each doubling of IgE was associated with abnormal tympanometric status (OR, 1.25; 95% CI, 1.03–1.52; *p* = 0.023; Supplementary Table S2).

In exploratory component-specific analyses using type A tympanograms as the reference, IgE > 60 IU/mL was not significantly associated with a type B tympanogram (adjusted OR, 1.89; 95% CI, 0.61–5.79; *p* = 0.268) but was associated with a type C tympanogram (adjusted OR, 5.61; 95% CI, 2.02–15.57; *p* = 0.001). The continuous-IgE analyses showed the same pattern: each doubling of serum total IgE was not significantly associated with a type B tympanogram (adjusted OR, 1.18; 95% CI, 0.90–1.56; *p* = 0.229) but was associated with a type C tympanogram (adjusted OR, 1.31; 95% CI, 1.04–1.64; *p* = 0.021; Supplementary Table S3).

Excluding the two patients younger than 18 years, the principal findings were materially unchanged: tympanic membrane closure was 60/67 (89.6%) versus 95/97 (97.9%) in the IgE > 60 and ≤60 IU/mL groups, respectively (*p* = 0.033), and abnormal tympanometric status was 28/60 (46.7%) versus 22/88 (25.0%) (*p* = 0.008). A 6-month ABG ≤ 20 dB remained nonsignificant between groups (54/63 [85.7%] vs. 86/93 [92.5%], *p* = 0.189). In the adult-only multivariable model (*n* = 145), IgE > 60 IU/mL remained associated with abnormal tympanometric status (OR, 3.23; 95% CI, 1.45–7.23; *p* = 0.004; Supplementary Table S1).

## 4. Discussion

The main finding of this retrospective cohort is that preoperative serum total IgE was associated with abnormal tympanometric status 6 months after type I tympanoplasty in both the prespecified categorical analysis and the separate continuous analysis. Poor mastoid pneumatization was the other independent factor. A lower tympanic membrane closure rate was also observed in the IgE > 60 IU/mL group, whereas the 6-month ABG endpoint did not differ significantly. These findings reveal an association with this laboratory measure. Importantly, they do not prove clinically useful predictive or prognostic performance, clinically relevant allergic disease, or a causal mechanism driven by allergy. The 60 IU/mL cutoff was the upper reference limit of the institutional assay and was not validated as a threshold for postoperative risk stratification. Total serum IgE has limited specificity for clinically relevant allergy, and reference intervals vary according to age, population, and assay [23]. Moreover, the present dataset did not include the clinical information required to define allergic rhinitis or active upper-airway allergic inflammation, including nasal symptoms, allergen-specific testing, nasal examination findings, or antiallergic treatment status.

The component-specific analysis further refined the interpretation of the primary endpoint. The association between serum total IgE and abnormal postoperative tympanometric status was driven primarily by the greater frequency of type C tympanograms with measurable negative-pressure peaks in the higher-IgE group, whereas no clear association was observed for type B tympanograms. This pattern supports a specific association between serum total IgE and postoperative middle-ear pressure dysregulation rather than abnormal tympanometric status of all types. However, these data cannot confirm that elevated serum total IgE gives rise to postoperative negative pressure, nor can we state that this association arises from clinically meaningful allergic inflammation. In addition, the component-specific analyses were limited by small sample sizes, with merely 19 type B and 32 type C events; these models should thus be interpreted cautiously as exploratory.

Previous studies have mainly focused on local and structural variables, including perforation size, ET function, middle-ear mucosal condition, mastoid pneumatization, graft material, and surgeon-related factors. A recent systematic review and meta-analysis by Illés and colleagues highlighted the multifactorial nature of tympanic membrane reconstruction, identifying age, perforation size, contralateral ear status, and surgeon experience as significant predictors, whereas other commonly discussed factors, including ear discharge, perforation site, previous adenoid surgery, and smoking, were not consistently associated with surgical success [5]. Our findings are in line with this broader concept that postoperative outcomes are not determined by a single local factor. In the present cohort, the association between serum total IgE and postoperative tympanometric status should be regarded as exploratory. Preoperative ETD was not independently associated with the 6-month tympanometric endpoint after adjustment, whereas poor mastoid pneumatization showed a strong association. This finding is consistent with the proposed pressure-buffering and ventilation role of the mastoid air-cell system [24,25,26]. The absence of an independent association for a single preoperative ETS category should not be interpreted as evidence that Eustachian tube function is unimportant; rather, a categorical preoperative score may not fully capture postoperative middle-ear ventilation.

The relationship between ETD and tympanoplasty outcome is also complex. Recent studies have yielded mixed findings regarding the prognostic value of eustachian tube function in type I tympanoplasty. Some prospective studies reported that impaired preoperative tubal function was associated with poorer graft or hearing outcomes [9,27,28], whereas recent studies in patients with chronic perforation and Eustachian tube dysfunction showed that satisfactory graft uptake could still be achieved, particularly when cartilage grafting was used [29,30]. This inconsistency may reflect differences in ETD definitions, measurement tools, disease activity, and endpoint selection. In the present study, preoperative ETD was not an independent predictor of abnormal postoperative tympanometric status after adjustment. This does not mean that tubal function is unimportant. Rather, it suggests that a single preoperative ETD classification may not fully capture postoperative pressure recovery after tympanic membrane reconstruction.

Previous studies linking clinically defined allergic rhinitis to eustachian tube dysfunction provide relevant clinical context but should be distinguished from the serum total IgE measure evaluated in the present study. The recent EAACI position paper recognizes a potential contribution of IgE-mediated immune mechanisms to eustachian tube dysfunction and middle-ear disease, while emphasizing that the available evidence remains heterogeneous and sometimes conflicting [18]. Chen et al. reported that concomitant allergic rhinitis was associated with failure of ETDQ-7 normalization after endoscopic sinus surgery in patients with chronic rhinosinusitis [31]. However, tympanoplasty-specific evidence is limited and inconsistent. Callioglu et al. observed a numerically lower graft success rate in patients with allergic rhinitis than in those without allergic rhinitis, but the difference was not statistically significant [32]. More recently, Çallıoğlu et al. found no increased graft failure among patients with treated allergic rhinitis undergoing type I cartilage tympanoplasty [33]. Taken together, these studies suggest a possible relationship between clinically defined upper-airway allergic disease and eustachian tube or middle-ear function, but they do not establish that elevated serum total IgE itself causes adverse tympanoplasty outcomes. Therefore, the association observed in our cohort should be interpreted as an association with a laboratory measure; whether it reflects allergic inflammation, another immunologic phenotype, or residual confounding cannot be determined from the present data.

The audiometric findings require separate interpretation. Audiometric outcomes after tympanoplasty are influenced by multiple factors, including ossicular status, graft mechanics, cochlear reserve, preoperative hearing level, and postoperative aeration [9,34]. In the present cohort, the proportion of ears with a 6-month ABG ≤ 20 dB did not differ significantly between serum total IgE categories. Among ears with a preoperative ABG > 20 dB, the proportion reaching an ABG ≤ 20 dB at 6 months was also similar between groups. In contrast, both the prespecified categorical analysis and the separate continuous-IgE sensitivity analysis supported an association with abnormal postoperative tympanometric status. The exploratory component analysis suggested that this association was concentrated in type C tympanograms with measurable negative-pressure peaks rather than type B tympanograms. These findings suggest that the observed IgE association was more evident for tympanometric status than for the audiometric endpoint, although the underlying mechanism remains uncertain. Poor mastoid pneumatization was the other independent predictor of abnormal postoperative tympanometric status [24]. The mastoid air-cell system may act as a pressure buffer for the middle ear, and poorer pneumatization may reduce the capacity to maintain stable aeration after tympanoplasty [25,26,35]. Poor mastoid pneumatization may therefore represent a structural limitation in postoperative middle-ear pressure regulation independent of the serum total IgE association.

The findings also inform interpretation of adjunctive eustachian tube procedures. Concomitant balloon dilation was more frequent in the IgE > 60 IU/mL group but was not independently associated with postoperative tympanometric status after adjustment. This finding is consistent with a recent randomized clinical trial showing no clear additional benefit of adding Eustachian tube balloon dilation to tympanoplasty in chronic inflammatory middle-ear disease with obstructive ETD [36].

Several limitations should be acknowledged. First, this was a retrospective single-center study, and residual confounding cannot be excluded. Second, the primary exposure used an assay-specific binary threshold. Although the separate analysis of log2-transformed continuous IgE supported a graded association, the functional form of the relationship and the clinical utility of alternative thresholds were not evaluated. Third, clinically defined allergic disease and antiallergic treatment status were not available, so the study cannot address whether allergic rhinitis itself influences tympanoplasty outcome. Fourth, postoperative endoscopic, tympanometric, and audiometric data were incomplete in some patients. Fifth, the multivariable model was based on complete cases and a limited number of abnormal tympanometric outcomes, so the adjusted estimates should be interpreted cautiously. Sixth, wet-ear status was heterogeneous because purulent and nonpurulent secretion were not consistently distinguished and microbiological culture was not routinely performed. Seventh, the component-specific models included small-number outcomes and were exploratory; their estimates may be unstable and require confirmation in larger cohorts.

Future prospective studies should confirm the association between serum total IgE and postoperative tympanometric outcomes and determine whether IgE provides incremental predictive information beyond established otologic factors. Such studies should use prespecified models with appropriate assessment of discrimination, calibration, and external validity. They should also concurrently characterize clinically defined allergic rhinitis, allergen-specific IgE, nasal symptoms, antiallergic treatment, and middle-ear inflammatory phenotype to determine whether any serum-IgE association is related to clinically relevant allergic inflammation. Future prospective studies should retain exact numerical tympanometric peak pressures.

## 5. Conclusions

Preoperative serum total IgE was associated with abnormal tympanometric status at 6 months after type I tympanoplasty in both categorical and continuous analyses. Exploratory component-specific analyses suggested that this association was concentrated in type C tympanograms with measurable negative-pressure peaks rather than type B tympanograms. A lower unadjusted tympanic membrane closure rate was also observed in the IgE > 60 IU/mL group, whereas the audiometric endpoint did not differ significantly. The 60 IU/mL cutoff was an assay-specific reference limit rather than a validated postoperative risk threshold. These findings describe an association with a laboratory measure and do not establish clinically useful predictive or prognostic performance, clinically defined allergy, or an allergy-mediated causal mechanism.

## Figures and Tables

**Table 1:** Clinical Characteristics According to Preoperative Serum Total IgE.

Variable	IgE ≤ 60 IU/mL (*n* = 101)	IgE > 60 IU/mL (*n* = 70)	*p* Value
Age, y	41.46 ± 13.68	45.23 ± 14.08	0.083
Female sex	63/101 (62.4)	36/70 (51.4)	0.161
Right ear	45/101 (44.6)	35/70 (50.0)	0.534
Wet ear	35/101 (34.7)	24/70 (34.3)	1.000
Large perforation	30/101 (29.7)	15/70 (21.4)	0.290
Preoperative ETD (ETS ≤ 5)	53/101 (52.5)	38/70 (54.3)	0.877
Poor mastoid pneumatization	38/100 (38.0)	26/68 (38.2)	1.000
Concomitant balloon dilation	25/101 (24.8)	28/70 (40.0)	0.044
Preoperative ABG > 20 dB	41/100 (41.0)	26/69 (37.7)	0.749
Preoperative air-conduction PTA, dB	40.69 ± 15.97	40.10 ± 17.73	0.826
Preoperative bone conduction, dB	20.42 ± 13.14	20.62 ± 11.98	0.917
Preoperative ABG, dB	20.36 ± 9.62	19.58 ± 9.89	0.611

Values are presented as mean ± SD or *n*/N (%). ETD, Eustachian tube dysfunction; PTA, pure-tone average; ABG, air-bone gap.

**Table 2:** (**A**). Overall Surgical Outcomes. (**B**) Preoperative and 6-month postoperative audiometric measures.

**(A)**			
**Outcome**			**Value**
Tympanic membrane closure			157/166 (94.6)
Normal postoperative tympanometric status			99/150 (66.0)
Abnormal postoperative tympanometric status			51/150 (34.0)
6-month ABG ≤ 20 dB			141/158 (89.2)
**(B)**			
**Variable**	**Preoperative**	**Postoperative**	***p*** **Value**
Air-conduction PTA, dB	40.03 ± 16.63	30.96 ± 14.87	<0.001
Bone conduction, dB	20.20 ± 12.51	18.41 ± 11.58	<0.001
ABG, dB	19.92 ± 9.55	12.58 ± 7.30	<0.001

Note A: Values are presented as *n*/N (%). All outcomes were assessed at 6 months. Normal tympanometric status was defined as a measurable peak pressure ≥ −50 daPa; abnormal status was defined as a measurable peak pressure < −50 daPa or a type B tympanogram. Note B: Values are presented as mean ± SD. *p* values were calculated using paired t tests. PTA, pure-tone average; ABG, air-bone gap.

**Table 3:** Six-Month Outcomes According to Preoperative Serum Total IgE Category.

Outcome	IgE ≤ 60 IU/mL	IgE > 60 IU/mL	*p* Value
Tympanic membrane closure	96/98 (98.0)	61/68 (89.7)	0.033
Abnormal postoperative tympanometric status	22/89 (24.7)	29/61 (47.5)	0.005
6-month postoperative tympanogram			0.009
Type A	67/89 (75.3)	32/61 (52.5)	
Type B	10/89 (11.2)	9/61 (14.8)	
Type C, peak pressure < −50 daPa	12/89 (13.5)	20/61 (32.8)	
6-month ABG ≤ 20 dB	87/94 (92.6)	54/64 (84.4)	0.121
6-month ABG ≤ 20 dB among ears with preoperative ABG > 20 dB	32/38 (84.2)	19/25 (76.0)	0.517

Values are presented as *n*/N (%). The *p*-value for the three-category postoperative tympanogram distribution was calculated using the Pearson chi-square test. Type C was defined as a measurable peak pressure below −50 daPa. Twenty-one ears without available 6-month tympanometry were excluded from the tympanometric analyses.

**Table 4:** Multivariable Logistic Regression Analysis for Abnormal 6-Month Postoperative Tympanometric Status.

Variable	Odds Ratio	95% CI	*p* Value
Age, per year	1.00	0.98–1.03	0.743
Female sex	0.89	0.40–1.99	0.783
Wet ear	1.30	0.56–3.05	0.541
Large perforation (≥50%)	1.44	0.58–3.58	0.437
Preoperative ETD (ETS ≤ 5)	1.82	0.73–4.54	0.198
Poor mastoid pneumatization	4.68	2.15–10.21	<0.001
IgE > 60 IU/mL	3.46	1.56–7.70	0.002
Concomitant balloon dilation	0.63	0.23–1.73	0.372

Complete-case analysis included 147 ears. The dependent variable was abnormal postoperative tympanometric status at 6 months, defined as a measurable peak pressure < −50 daPa or a type B tympanogram. CI, confidence interval; ETD, Eustachian tube dysfunction; ETS, Eustachian Tube Score.

## Data Availability

The data presented in this study are available from the corresponding author upon reasonable request. The dataset has not yet been deposited in a public repository.

## Supplementary Materials

The following supporting information can be downloaded at https://www.mdpi.com/article/10.3390/audiolres16050146/s1: Table S1: Adult-Only Sensitivity Analysis (Age ≥ 18 Years); Table S2: Multivariable Logistic Regression Treating Serum Total IgE as a Continuous Variable; Table S3: Exploratory Component-Specific Logistic Regression Analyses of 6-Month Postoperative Tympanogram Type.
