# Supplementary material for: Preoperative Serum Total IgE Category and 6-Month Tympanometric Status After Type I Tympanoplasty for Chronic Otitis Media
Source: Audiol Res. 2026 Sep 28;16(5):146. doi: 10.3390/audiolres16050146 (PMC13701292; doi:10.3390/audiolres16050146)
Supplement: Supplementary file 1 [file audiolres-16-00146-s001.zip › audiolres-4561920-supplementary.pdf]

Preoperative Serum Total IgE Category and 6-Month Tympanometric Status After Type I Tympanoplasty for Chronic Otitis Media

Supplementary Table S1. Adult-Only Sensitivity Analysis (Age ≥18 Years)

Outcome	IgE ≤60 IU/mL	IgE >60 IU/mL	P value
Tympanic membrane closure	95/97 (97.9)	60/67 (89.6)	0.033
Abnormal postoperative tympanometric status	22/88 (25.0)	28/60 (46.7)	0.008
6-month ABG ≤20 dB	86/93 (92.5)	54/63 (85.7)	0.189

Values are presented as n/N (%). Two patients younger than 18 years (ages 8 and 15 years) were excluded. In the adult-only multivariable logistic regression model (n=145), serum total IgE >60 IU/mL remained associated with abnormal postoperative tympanometric status (OR, 3.23; 95% CI, 1.45–7.23; P=0.004).

Supplementary Table S2. Multivariable Logistic Regression Treating Serum Total IgE as a Continuous Variable

Variable	Odds ratio	95% CI	P value
Age, per year	1.01	0.98–1.03	0.698
Female sex	0.92	0.42–2.04	0.842
Wet ear	1.21	0.53–2.78	0.647
Large perforation (≥50%)	1.47	0.59–3.63	0.407
Preoperative ETD (ETS ≤5)	1.69	0.69–4.16	0.255
Poor mastoid pneumatization	4.72	2.19–10.16	<0.001
Serum total IgE, per doubling	1.25	1.03–1.52	0.023
Concomitant balloon dilation	0.75	0.28–2.01	0.573

Complete-case analysis included 147 ears. Serum total IgE was log2-transformed; therefore, the odds ratio represents each doubling in IgE concentration. The dependent variable was abnormal postoperative tympanometric status at 6 months, defined as a measurable peak pressure <−50 daPa or a type B tympanogram.CI, confidence interval; ETD, Eustachian tube dysfunction; ETS, Eustachian Tube Score.

Supplementary Table S3. Exploratory Component-Specific Logistic Regression Analyses of 6-Month Postoperative Tympanogram Type

Outcome comparison	Serum total IgE variable	Unadjusted OR (95% CI)	P value	Adjusted OR (95% CI)	P value
**Type B vs type A**	IgE >60 vs ≤60 IU/mL	1.88 (0.70–5.09)	0.212	1.89 (0.61–5.79)	0.268
	IgE, per doubling	1.14 (0.89–1.47)	0.308	1.18 (0.90–1.56)	0.229
**Type C vs type A**	IgE >60 vs ≤60 IU/mL	3.49 (1.52–8.01)	0.003	5.61 (2.02–15.57)	0.001
	IgE, per doubling	1.21 (0.99–1.48)	0.058	1.31 (1.04–1.64)	0.021

OR, odds ratio; CI, confidence interval; IgE, immunoglobulin E; ETD, Eustachian tube dysfunction.
